# What Are the Cleaning and Disinfection Methods for Acrylic Orthodontic Removable Appliance? A Systematic Review

**DOI:** 10.3390/children8110967

**Published:** 2021-10-26

**Authors:** Carole Charavet, Léa Graveline, Zoé Gourdain, Laurence Lupi

**Affiliations:** 1 Université Côte d’Azur, Faculté de Chirurgie Dentaire, 06800 Nice, France; lea.graveline@etu.univ-cotedazur.fr (L.G.); zoe.gourdain@etu.univ-cotedazur.fr (Z.G.); Laurence.LUPI@univ-cotedazur.fr (L.L.); 2 Centre Hospitalier Universitaire de Nice, Pôle Odontologie, 06000 Nice, France; 3 Laboratoire MICORALIS UPR 7354, Université Côte d’Azur, 06800 Nice, France

**Keywords:** removable orthodontic appliances, biofilm, cleaning methods, disinfection methods

## Abstract

(1) Background: The use of removable orthodontic appliances, which is common in early ages, requires careful hygiene, as several different microorganisms are found on their surface during the orthodontic treatment. (2) Methods: Comprehensive electronic searches were conducted up to May 2021. Randomized controlled trials (RCTs) or controlled clinical trials (CCTs), prospective or retrospective, evaluating the efficacy of cleaning and disinfection methods for acrylic removable orthodontic appliances, redacted in the English language, were included. Three independent blinding review authors were involved in study selection, data extraction, and bias assessment. (3) Results: A total of 2491 records were screened and eight studies (six RCTs and two CCTs) fulfilled the inclusion criteria. Among the overall cleaning and disinfection methods described in the included studies, four categories could be defined: liquid antimicrobial agents, commercial tablet cleansers, natural plant extracts and incorporation of quaternary ammonium methacryloxy silicate, all of which demonstrated superior efficacy compared to the placebo/negative control. However, the different methods were not compared with each other. (4) Conclusions: Biofilm control on acrylic orthodontic removable appliances can be performed using the different cleaning and disinfection methods considered in the included studies. Further studies are needed to define the most effective technique. Registration: PROSPERO CRD 42021269297.

## 1. Introduction

Oral hygiene has always been a challenge in orthodontics and the relation between orthodontic treatments and caries development, or periodontal diseases has been well studied [1,2]. Orthodontic treatment induces several modifications of the oral environment, including a decrease in salivary pH [3], a facilitation of dental biofilm adherence on the orthodontic surface appliance, and an increase in cariogenic (*S. mutans*, *Lactobacillus* sp.) and periodontal pathogenic microorganisms levels (*T. denticola*, *A. actinomycetemcomitans*) [4,5,6,7]. In addition, children represent a large amount of the orthodontic population, in which oral hygiene compliance is not perfect, associated with a possible lack of dexterity. Hence, hygiene methods in patients undergoing orthodontic treatment with fixed appliances have been widely addressed in the literature, whether it was brushing method [8] possibly assisted with smartphone application [9], use of mouthwashes [10], or direct use of antimicrobial agents in brackets, wires and orthodontic adhesives [11,12].

Orthodontic treatments by acrylic removable orthodontic appliance, mainly constituted with PolyMethylMethAcrylate (PMMA), are also concerned. Firstly, patients treated by removable appliances demonstrated proliferation of *C. albicans* salivary levels, responsible of increasing the risk of candidiasis and stomatitis [13]. Furthermore, biofilm developing directly on acrylic resin removable appliances contains a majority of non-streptococci anaerobic bacteria, *Streptococcus* spp., *Enterobacteriaceae*, and *Lactobacillus* spp. [14]. Recently, Rodriguez-Renteria et al. [15] investigated after 4 weeks the frequency of *Staphylococcus aureus*, *Pseudomonas aeruginosa*, and *Candida* species on removable orthodontic appliances and found that, of the 55 removable orthodontic appliances studies, *Staphylococcus aureus* was present on 90.9%, *Pseudomonas aeruginosa* on 67.7% and *Candida* species on 32.7% of the appliances. Interestingly, these microorganisms were also found in the support oral mucosa of the 55 children (respectively 89.09%; 60%; 30.9%). The authors also concluded a direct relationship between removable orthodontic treatment and an increase in the amount of periodontal pathogenic microorganisms. Surface roughness, incomplete polymerization, or wear caused by daily brushing of the appliance seems to directly influence bacterial [16,17] and fungal [18] biofilm adhesion to removable acrylic appliances.

In addition, orthodontic treatment by removable acrylic appliance involves not only perfect oral hygiene maintenance but also a cleaning protocol for the appliance itself. Some studies have already been conducted in order to investigate the effects of different cleaning protocols for acrylic removable orthodontic appliances and may also help to reduce the risk of oral diseases related to biofilm growth. Indeed, some different processes have been described such as denture cleaners, enzymatic solutions, chlorhexidine, sodium hypochlorite, or “homemade” solutions containing vinegar or citric acid [19]. Additionally, professional methods performed by orthodontists, such as ultrasounds, have also been described [20]. However, to the best of our knowledge, no systematic review (SR) has been carried out on the subject.

Therefore, for the first time, the aim of this SR was to investigate the different methods of cleaning and disinfection for acrylic resin removable orthodontic appliances.

## 2. Materials and Methods

### 2.1. Protocol Registration

A systematic review was carried out following as closely as possible the guidelines of preferred reporting items for systematic reviews and meta-analyses (PRISMA) and recommendations [21]. The protocol was registered in the International Prospective Register of Systematic Review (PROSPERO) (CRD42021269297).

### 2.2. Focused Question and Eligibility Criteria

The research question of the present SR was stated as follows: “What are the cleaning and disinfection protocols for removable acrylic resin orthodontic appliances?”

Then, the definition of Population, Intervention, Comparison, and Outcome (PICO) was established upon the focused research question as follows:

− P (Population/Problem): Removable acrylic orthodontic appliances worn by orthodontic patients;
− I (Intervention): Cleaning and disinfection methods;
− C (Comparison): Placebo or negative control;
− O (Outcome): Global biofilm amount, *S. mutans* colony count or, *Candida* colony count on removable acrylic orthodontic appliances.

The inclusion criteria for admittance in the SR were defined as follows: Randomized Controlled Trials (RCTs) or nonrandomized trials (Controlled Clinical Trials; CCTs), prospective or retrospective, evaluating the efficacy of cleaning and disinfection methods for acrylic removable orthodontic appliances by comparing them with a placebo or negative control, redacted in the English language. There were no limitations regarding the publication date of the articles. The exclusion criteria consisted of non-comparative studies (case reports and case series), surveys, editorials, opinions, reviews, in vitro studies, animal model experimental studies, systematic reviews, or meta-analyses.

### 2.3. Search Strategy and Information Sources

A computerized search was conducted for the last time on 29 May 2021 without time restriction on several databases including PubMed, Scopus, Cochrane Central Register of Controlled Trials (CENTRAL), and Embase in order to identify eligible published studies. A search strategy combining keywords, Medical Subject Headings (MeSH) terms and Boolean operators was done and then adapted for each database (Table 1). Search on the Scopus database was limited to articles regarding dentistry. Additionally, a manual search was performed in the bibliography of each included paper in order to identify articles that were not found by the electronic search.

### 2.4. Study Selection

To establish their eligibility, evaluation of the selected articles was staged in a two-step progression after the removal of duplicate records between databases using a reference management software (Zotero version 5.0.96.2). First, the selection procedure was performed by reading the title and then the abstract, independently by two reviewers. Afterwards, a verdict for final eligibility was performed based on full-text assessment by the same reviewers. In case of disagreements, a third reviewer was consulted.

### 2.5. Data Collection Procedure

Data were extracted independently in duplicate by two reviewers from the included studies that satisfied the inclusion criteria, according to the PICO approach. The following information was extracted from each included paper: first author’s name, year of publication, country, study design, inclusion/exclusion criteria for subjects, description of intervention protocol, comparison, and results. As the included studies showed heterogenic methods, methodology analysis, and outcomes, performing a meta-analysis was not possible. Therefore, the analysis of the articles was qualitative and descriptive.

### 2.6. Risk of Bias Evaluation

The evaluations of risk of bias were performed by two reviewers independently and discordances in the results were resolved by discussion between the authors and, if necessary, a third reviewer was consulted.

The risk of bias in eligible RCTs was assessed following the “Cochrane’s collaboration tool for assessing the risk of bias in randomized trials” [22]. The assessment criteria contained seven items. A judgment score was provided following the recommendations of the Cochrane Handbook for Systematic Reviews of Interventions 6.0 (https://handbook.cochrane.org) for each bias domain.

Additionally, risk of bias in eligible CCTs was judged following the ROBINS-I (Risk of Bias in Non-randomized Studies-of Interventions) tool [23] containing seven domains, through which bias might be introduced, scored as low, moderate, serious, or critical risk of bias after answering the signaling questions following the Cochrane Handbook for Systematic Reviews of Interventions 6.0.

Risk-of-bias plots figures were produced using the robvis tool [24].

## 3. Results

### 3.1. Study Selection

**Figure 1:**
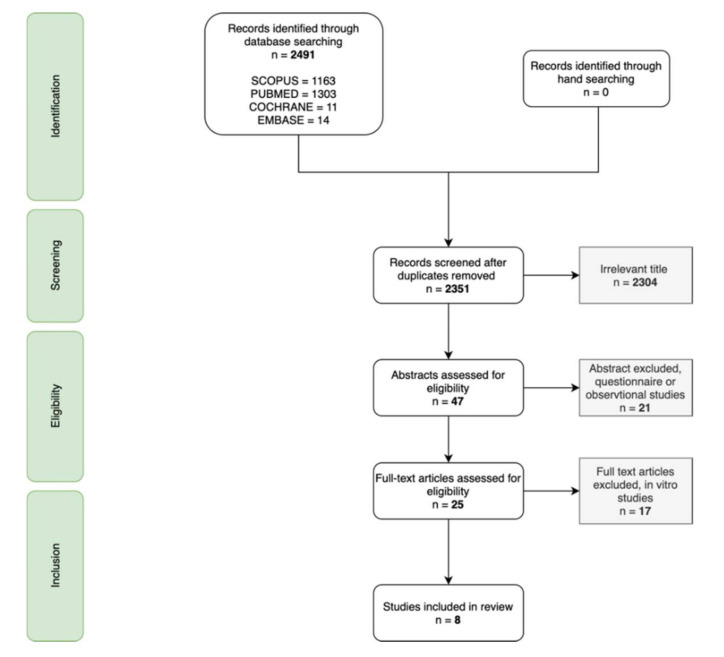
Preferred reporting items for systematic reviews and meta-analyses (PRISMA) flowchart.

The search strategy initially revealed 2491 records across the four databases: 140 duplicates were eliminated and 2304 records out of 2351 remaining were discarded based on their title and language. The 47 remaining articles were assessed on their abstract, in accordance with the eligibility criteria. Further evaluation was conducted on 25 full-text articles, which led to eight articles eligible for this systematic review. The flow diagram retracing the search strategy is detailed in Figure 1.

### 3.2. Study Characteristics

Characteristics for each individual study are described in Table 2 and Table 3. Out of the eight studies reviewed, six of them were randomized controlled trials [25,26,27,28,29,30] and two were non-randomized controlled studies [31,32], published from 2006 to 2020. In terms of geographic localization, three studies were conducted in Brazil [27,29,30], two in Malta [25,26], one in Germany [31], one in China and USA [28], and one in India [32]. All studies considered, a total of 318 subjects were enrolled in the studies, and also 318 acrylic removable orthodontic appliances were studied.

Three studies assessed the efficiency of liquid solution (enzymatic commercial solution (Ortoform^®^), 0.5% sodium hypochlorite, 0.05% cetylpyridinium chloride (Cepacol^®^) or 0.12% chlorhexidine gluconate (Periogard^®^)) [27,29,30], three used commercial cleaning tablets [25,26,31], one investigated the effects of natural plant extracts [32] and the remaining one [28] evaluated incorporating an antimicrobial agent in the resin.

Global biofilm amount, with no distinction between microbial species, was evaluated by four [28,29,31,32] of the studies. Two studies specifically counted *Mutans streptococci* (MS) colonies [27,30], while the remaining two focused on *Candida* amount (all subspecies included) [25,26].

### 3.3. Results of Bias

**Figure 2:**
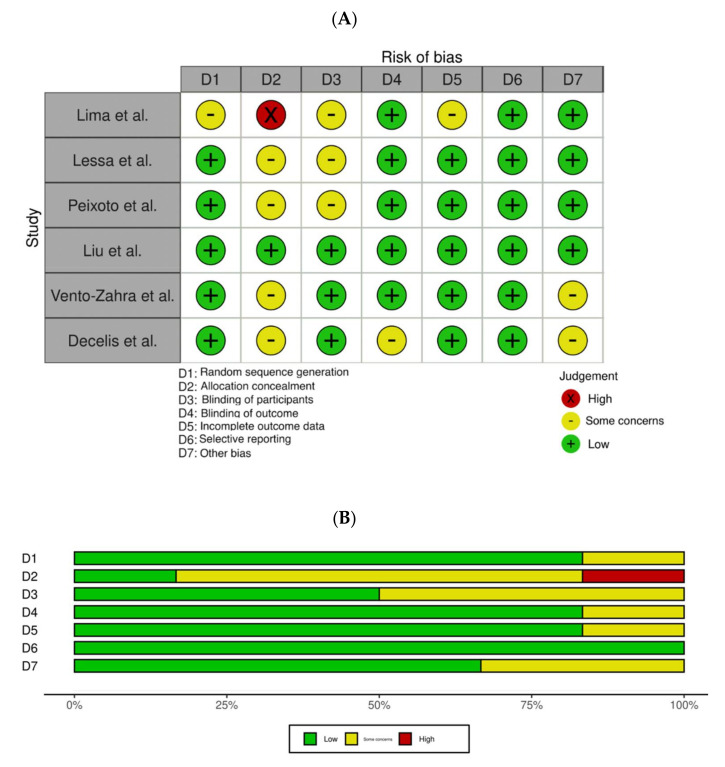
(**A**) Risk of bias assessment in randomized controlled trials (RCTs), according to the Cochrane Collaboration’s tool. (**B**) Summary of risk of bias per items from D1 to D7.

**Figure 3:**
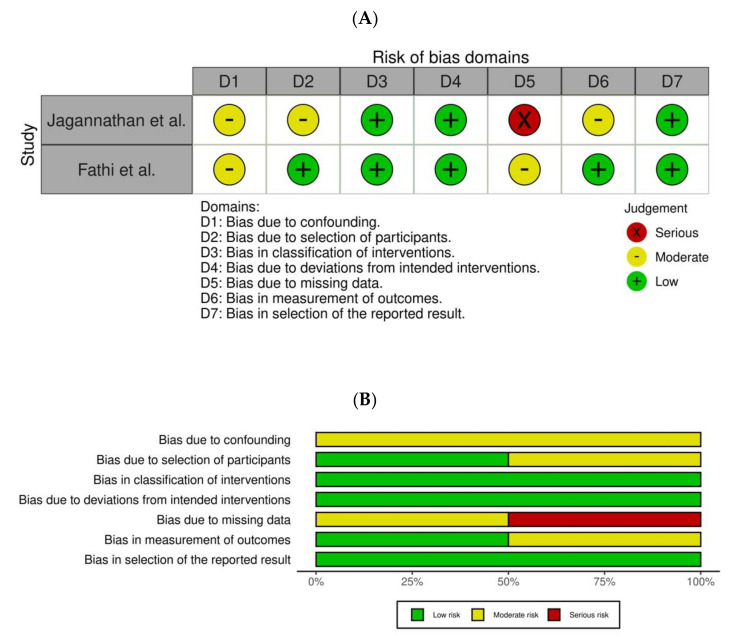
(**A**) Risk of bias assessment in controlled clinical trials (CCTs) according to the ROBINS-I assessment tool. (**B**) Summary of risk of bias per items from D1 to D7.

Risk-of-bias plots using the robvis tool [24] are available in Figure 2A,B and Figure 3A,B.

Amongst RCTs (Figure 2A,B), the study by Lima et al. [29] was identified as the one having the highest risk of bias. The study by Liu et al. [28] was reported as having the lowest risk of bias. Overall, the highest risk of bias was found on the allocation concealment followed by the blinding of participants. No study was found to have carried out selective reporting.

Amongst CCTs (Figure 3A,B), the study by Jagannathan et al. [32] was found to have the highest risk of bias out of the two. The highest risk of bias was found on the missing data, followed by the bias due to confounding.

### 3.4. Results of Individual Studies

All studies found significant differences between the control group and the intervention group. All results are described in Table 3.

Amongst liquid antimicrobial agents, Lessa et al. [30] found that chlorhexidine gluconate (Periogard^®^) was significantly more effective than cetylpyridinium chloride (Cepacol^®^); another study by Lima et al. [29] found sodium hypochlorite significantly more effective than the enzymatic solution (Ortoform^®^).

Regarding commercial tablet cleansers (NitrAdine^®^, Kukis^®^, and fittydent^®^) [25,26,31], all of them showed significant efficacy against control, being either a placebo or water. NitrAdine^®^ was the only one tested against a placebo tablet [25,26] and was proven to significantly decrease plaque index, *C. albicans* colony count, and odor of the appliance, but not to significantly decrease salivary levels of *Candida* spp.

Significant efficiency for several natural extracts (neem, katha, and cinnamon) was proven against negative control, normal saline [32], but none were found significantly as efficient as the most common agent used for acrylic baseplate disinfection, chlorhexidine.

The incorporation of a quaternary ammonium methacryloxy silicate in orthodontic PMMA resin has shown a significant increase in the amount of dead microorganisms in biofilm [28]. However, no significant decrease in biovolume was observed.

## 4. Discussion

The present study is the first systematic review (SR) performed on the topic of cleaning and disinfection methods for removable acrylic orthodontic appliances and four different categories of methods can be addressed and discussed.

Regarding the different liquid solutions used, chlorhexidine gluconate (Periogard^®^) and sodium hypochlorite were found to be more effective than cetylpyridinium chloride (Cepacol^®^) and the enzymatic solution (Ortoform^®^) respectively. However, these four solutions have not been compared to each other, making it impossible to discuss the superiority of one of them. Many of them are already commonly used in other domains, in which their effectiveness has already been demonstrated. Periogard^®^ is a mouthwash containing chlorhexidine gluconate as its main active ingredient, whose antimicrobial effect has already been proven, thus being widely used in treating periodontal diseases [33]. Cepacol^®^, used in the study [30] included in this SR, is a spray containing cetylpyridinium chloride, which is used as a treatment for sore throats [34] and its efficacy has been demonstrated in this specific disease but is still less effective than chlorhexidine gluconate for acrylic orthodontic appliances. Beyond the efficiency of products, Lessa et al. [30] specified that sprayed solutions seemed to be more practical and perhaps economical than other forms.

Sodium hypochlorite is a commonly relevant used active ingredient in dentistry, especially in endodontics for root canal disinfection [35]. Lima et al. showed its significant efficiency compared to Orthoform^®^ and no treatment. However, the smell and taste left by sodium hypochlorite may call into question the need to rinse it off the appliance after treatment, and its ability to whiten acrylic resin represents a disadvantage. The enzymatic solution (Ortoform^®^), on the other hand, did not show a significant difference with the control group (no treatment). As the proteolytic enzyme seem to be an interesting track to explore, it might be a need to increase the duration of immersion in order to have a significant antimicrobial effect. Nevertheless, this needs to considered with the wearing duration needed in order for removable orthodontic appliances to have their expected effect.

Concerning the commercial cleaning tablets, three different brands were assessed in this review: NitrAdine^®^ Ortho Junior [25,26,31], Fittydent^®^ [31], and Kukis^®^. NitrAdine^®^ and Kukis^®^ share the same active ingredient (citric acid) and similar excipients added to give the effervescent properties (sodium carbonate, sodium perborate), according to the manufacturers’ websites. Fittydent^®^ seems to have a close composition to the NitrAdine^®^ and Kukis^®^. All have demonstrated a significant effect on biofilm removal compared to water [31] or placebo [25,26]. Specifically, NitrAdine^®^ appears to have more of a bacteriostatic effect than a bactericidal effect on Candida [26]. Furthermore, according to Fathi et al. [31], whereas no demonstration was done to establish a significant association between exposure duration to active agent and cleaning results, a link in this direction could be suggested. Finally, as the placebo has an effervescent effect in the studies by Decelis et al. and Vento-Zahra et al. [25,26], the action of these tablets seems to be related to the chemical effects and not to the mechanical effects of the microbubbles produced by effervescent tablets.

Natural extracts [32] from cinnamon, neem and, katha demonstrated interesting abilities to decrease the amount of biofilm on removable acrylic appliances, but their efficacy remained significantly lower than chlorhexidine. These medicinal plants could be considered as natural disinfectant agents, as their positive effect has been already demonstrated in various medical fields [36]. As opposed to chlorhexidine, which can bring acrylic discoloration or staining, these extracts could be used with no side effects. Further investigation needs to be performed to precise this possible alternative to chemicals products currently used.

Regarding incorporated agents in resin such as the Quaternary Ammonium Methacryloxy Silicate (QAMS), Liu et al. [28] exhibited favorable antimicrobial activity by this agent against plaque biofilm. These results were in accordance with the in vitro study of Gong et al. [37,38], which found better mechanical properties in QAMS-containing resin with no significant cytotoxicity. Furthermore, they evaluated the long-term efficacy of the agent and found that the active ingredient remains stable over time. Other in vitro studies used different agents, such as UV-responsive photocatalyst coatings [39], nanoparticles [40,41,42,43] or extracts from plants or algae [44,45]. All of them have shown encouraging results on their antimicrobial properties, however, none of these provided in vivo results, which could therefore be an interesting track to explore.

If orthodontic practitioners’ advice to their patients is confronted with what has been shown in this present SR, there are discrepancies that should not be ignored. According to surveys conducted by Eichenauer et al. [19] and Tsolakis et al. [46], almost all orthodontists recommend mechanical cleaning with a toothbrush to clean removable orthodontic appliances. Moreover, a smaller portion recommended the associated use of chemical aids, mentioning denture cleaners, commercial disinfection solutions, vinegar, or citric acid diluted with water. Furthermore, vinegar has not been investigated by studies in this review, and its efficacy and long-term effects on resin structure have not been established. Despite significant evidence that associating manual brushing and a chemical cleaning agent is more efficient than brushing by itself [27,30], the majority of practitioners don’t recommend this solution.

Finally, some additional points cannot be ignored regarding this SR. First, disinfection protocols need to preserve the initial resin structure. However, only one of the included studies [29] mentioned the evolution of the appliances’ surface proprieties. Furthermore, as surface roughness could influence biofilm adherence, it would be interesting to work on this point in order to limit its adhesion. Moreover, although five studies relied on subjects themselves to perform all or part of the cleaning protocols [25,26,27,29,30], only Decelis et al. described clear criteria to assess compliance of patients in wearing the appliance. Furthermore, none of them described a way to evaluate the observance of subjects in cleaning protocols. This represents a limitation that is important to consider when studying at-home cleaning protocols. Surprisingly, no study has directly compared manual brushing alone with an antimicrobial agent alone. It seems interesting to have the effectiveness of manual brushing alone to get a sort of “baseline”. In any case, manual brushing seems to be an essential step. Finally, the heterogeneity among the different studies and the absence of comparison between the methods did not allow for precise guidelines. Conclusively, few studies were finally included in this SR, and, as Lessa et al. [30] pointed out, the subject is not well developed in orthodontics: do we realize how important contamination of our orthodontic removable acrylic appliances is?

## 5. Conclusions

Based on this systematic review, some cleaning and disinfection methods as categorized in liquid antimicrobial agents, commercial tablet cleansers, natural plant extracts, and incorporation of quaternary ammonium methacryloxy silicate have a positive impact to control or remove biofilm formation. However, as they have not been compared with each other, it remains impossible to know which one is the most effective. Further studies designed as randomized controlled trials are also very much needed to explore this topic.

## Figures and Tables

**Table 1:** Search strategy.

Database	Keywords
Pubmed	((“removable orthodontic appliance”) OR “Orthodontic Appliances, Removable”[MAJR] OR ((preventive OR functional) AND appliance AND orthod*) OR (orthod* resin) OR (orthod* acrylic)) AND (((removal OR reducing OR controlling) AND (biofilm OR bacteria OR “dental plaque”)) OR disinfection OR decontamination OR “Disinfection/methods”[MeSH] OR “Biofilms/drug effects”[MeSH] OR “Equipment Contamination/prevention and control”[Mesh] OR “Anti-Infective Agents, Local”[Mesh] OR “Oral Hygiene”[MeSH] OR antimicrobial OR antibacterial OR clean*)
Scopus	((“removable orthodontic appliance”) OR ((preventive OR functional) AND appliance AND orthod*) OR (orthod* resin) OR (orthod* acrylic)) AND (((removal OR reducing OR controlling) AND (biofilm OR bacteria OR dental plaque)) OR decontamination OR disinfection OR antimicrobial OR antibacterial OR clean*) AND (LIMIT-TO (SUBJAREA,”DENT”)) AND NOT (fixed OR bracket)
Embase, Cochrane	(“removable appliance” AND “orthodontic”) AND (“biofilm removal” OR “biofilm adhesion” OR “decontamination” OR “disinfection” OR “dental plaque” OR “antimicrobial” OR “antibacterial” OR “clean*”)

**Table 2:** Data extracted from eligible records using the PICO approach. N/A = not applicable; RCT = randomized controlled trial; CCT = controlled clinical trial; ROA = removable orthodontic appliance; QAMS = quaternary ammonium methacryloxy silicate; PMMA = PolyMethylMethAcrylate; AJODO = american journal of orthodontics and dentofacial orthopedics.

Authors	Journal and Year Published	Study	Population	Inclusion and Exclusion Criteria	Intervention	Comparison	Support
Lima et al. [29]	Journal of Oral Rehabilitation, 2006	RCT	13 participants	N/A	0.5% NaOCl solution or enzymatic solution (Ortoform^®^)	Sterile tap water	None
Lessa et al. [30]	AJODO, 2007	RCT	20 participants	No use of antimicrobial mouthwash, no systemic disease and no use of antibAliotics in the previous 3 months	Antimicrobial sprays: Periogard or Cepacol	Sterile tap water	None
Peixoto et al. [27]	AJODO, 2011	RCT	21 participants	Good general health, favorable dental alignment and salivary counts of *S. mutans*. No use of antimicrobial mouthwash, no systemic disease and no use of antibiotics in the previous 3 months	Periogard once or twice a week and manual brushing of appliance	Sterile tap water and manual brushing of appliance	None
Liu et al. [28]	Scientific Reports, 2016	RCT	32 participants	Good general health, no active caries or periodontal disease. No use of antimicrobial mouthwash, no use of antibiotics in the previous 6 months, no cleft palate or gag reflex.	QAMS-containing PMMA orthodontic resin	Conventional PMMA orthodontic resin	None
Fathi et al. [31]	Journal of Orofacial Orthopedics, 2015	CCT	20 participants	No dentures or orthodontic appliances, no missing teeth, no active caries or periodontal disease	Fittydent ^®^, NitrAdine ^®^ or Kukis ^®^	Water	None
Jagganathan et al. [32]	Int Journal of Clinical Pediatric Dentistry, 2020	CCT	50 participants	Children in the mixed dentition using maxillary orthodontic appliances. Children not regularly visiting appointments or in the primary/permanent dentition were excluded.	Neem extract, katha extract and cinnamon extract	Chlorhexidine (*positive control*) and normal saline (*negative control*)	None
Vento-Zahra et al. [25]	Quintessence International, 2011	RCT	70 participants	Ongoing maxillary removable appliance therapy for more than 1-month, full time maxillary appliance wear, aged 11 to 14.	Nitradine^®^ tablets	Placebo	Yes
Decelis et al. [26]	Quintessence International, 2012	RCT	92 participants	Orthodontic treatment for at least a month, aged 11 to 14. No conditions or undergoing treatment predisposing to oral candidosis.	Nitradine ^®^ tablets	Placebo	Yes

**Table 3:** Outcomes of included studies. SEM = scanning electron microscopy; CLSM = confocal laser scanning microscopy.

Authors	Protocol	Variable (Main)	Results
Lima et al. [29]	Three phases of 4 days each, one for each treatment, with 3 different appliances of the same type.	Surface roughness and biofilm accumulation (light absorption at 280 nm)	No significant difference in surface roughness after treatment between Orthoform^®^, NaOCl and water (Control 0.497 mm; Orthoform^®^ 0.535 mm; NaOCl 0.496 mm; *p* > 0.05).
	Immersion eight times a day (5 min) in 20% sucrose solution to enhance biofilm formation.		In terms of biofilm accumulation, NaOCl was more efficient than control (Control: 0.547; NaOCl: 0.473; *p* < 0.05), but no difference was found between control and enzymatic solution (Control: 0.547; Orthoform^®^: 0.521; *p* > 0.05).
	Daily immersion in 3 solutions: sterile tap water (negative control); enzymatic commercial solution (Ortoform^®^) (30 min) or 0.5% sodium hypochlorite (NaOCL) (10 min).		
Lessa et al. [30]	A 3-stage changeover system with a 1-week interval between each stage (each stage being associated with a different solution).	*S. mutans* colony count (*SEM examination*)	Comparing *S. mutans* colony counts on appliances, both treatments were found significantly more efficient than control (*p* < 0.001). Periogard was found to be significantly more efficient than Cepacol (*p* < 0.001).
	Acrylic baseplate worn full time for 7 days except during meals, brushed once a day with toothbrush and toothpaste used by the patient. The baseplate was retrieved after one week and sprayed on both sides following a randomized protocol with either 0.05% cetylpyridinium chloride solution (Cepacol), 0.12% chlorhexidine gluconate solution (Periogard), or sterile tap water. Baseplates were then placed in an individual sterile receptacle containing a selective enrichment broth for *S. mutans* and were incubated for 3 to 4 days at 37 °C.		
Peixoto et al. [27]	Three-stage changeover system with a 1-week interval, each stage being associated with a different protocol. Acrylic baseplates were worn full time for 7 days except during meals, brushed thrice a day using a toothbrush and toothpaste. At bedtime, subjects were told to spray their appliances either: every day with sterile tap water (protocol I), on the 7th day with Periogard (protocol II), or on the 4th and 7th day with Periogard (protocol III). Baseplates were then placed in an individual sterile receptacle containing a selective enrichment broth for *S. mutans* and were incubated for 3 to 4 days at 37 °C.	*S. mutans* colony count (*SEM examination*)	Both protocols were significantly more efficient than control (percentage of *S. mutans* contaminated baseplates: water (protocol I) 100%; protocol II 80%; protocol III 60%; *p* < 0.05), but no significant difference was found between the two treatment protocols (*p* > 0.05).
Liu et al. [28]	Subjects were randomized into two groups: one whose appliance would have the QAMS-modified resin on the right side, and one who would have it on the right side (the other side being the control unmodified acrylic resin). The appliance was worn for 48 h continuously by subjects, then were retrieved for analysis of the resin disks. Biovolume was analyzed to determine the percentage of kill within the mass.	Percentage biofilm kill (*CLSM examination*)	QAMS had a significantly higher percentage kill than control disks (percentage kill in biovolume: control: 3.73 ± 2.11%; QAMS: 33.94 ± 22.88%; *p* ≤ 0.001).
Fathi et al. [31]	Splint was worn continuously for 96 h, taken off only for toothbrushing. They were then collected and randomized for cleaning protocol, which was either: immersion in fittydent super^®^ for 30 min, in Kukis^®^ for 10 min, in NitrAdine^®^ for 15 min, or in water (control) for 10 min. The protein amount on the resin surface was calculated before and after cleaning.	Amount of biofilm (*relative protein rate removal*)	Comparing relative median protein rate removal, Fittydent was found significantly more efficient than Kukis (Fittydent: 86.8%; Kukis: 79.8%; *p* = 0.001), but no other significant difference was found between tablets (fittydent^®^ vs. Nitradine^®^: 86.8% vs. 81.8%; *p* = 0.057; Nitradine^®^ vs. Kukis^®^: 81.8% vs. 79.8%; *p* = 0.411). Tablets were all significantly more efficient than water (median protein removal rate for water: 56.6%; *p* < 0.003).
Jagganathan et al. [32]	Salivary swabs were collected from palatal side of the appliance. Neem, katha, and cinnamon extracts were prepared. Samples were inoculated on agar plates, and antimicrobial agents were introduced with a micropipette	Zone of inhibition of extracts in saliva cultures	According to the mean zone of inhibition in saliva cultures, all extracts were more significantly efficient than negative control and less than positive control (*p* < 0.001). Neem and katha extract were significantly more efficient than cinnamon extract (*p* < 0.001).
Vento-Zahra et al. [25]	Patients were randomized into two groups: experimental (NitrAdine^®^ OrthoJunior) and control (placebo tablet). The appliance was treated by daily soaking in a solution containing the dissolved tablet and brushed with only water, for 6 weeks. Appliances were inspected visually before and after treatment, and saliva samples were collected and analyzed before and after treatment.	Salivary *Candida* levels	NitrAdine tablets significantly decreased significantly plaque index (*p* = 0.0253) and odor (*p* = 0.0007) of the appliance compared to the control group. However, no significant difference was found in salivary *Candida* levels between the two groups.
		Plaque index on the appliance	
		Appliance odor	
Decelis et al. [26]	Patients were randomized into two groups: experimental (NitrAdine^®^ OrthoJunior) and control (placebo tablet). The appliance was treated by daily soaking for 20 min in a solution containing the dissolved tablet and brushed with only water, for 6 weeks. Samples were taken on the palatal side of the appliance before and after treatment.	*Candida* colony count	Significant increase in *Candida* levels for control group during treatment (*p* = 0.008). A decrease, but not significant, of *Candida* levels during treatment for experimental group was found (*p* = 0.353).

## Data Availability

The data underlying this article are available in the article.
